# The Association of Peri-Procedural Blood Transfusion with Morbidity and Mortality in Patients Undergoing Percutaneous Lower Extremity Vascular Interventions: Insights from BMC2 VIC

**DOI:** 10.1371/journal.pone.0165796

**Published:** 2016-11-11

**Authors:** Peter K. Henke, Yeo Jung Park, Sachinder Hans, Paul Bove, Robert Cuff, Andris Kazmers, Theodore Schreiber, Hitinder S. Gurm, P. Michael Grossman

**Affiliations:** 1 Department of Surgery, University of Michigan, Ann Arbor, MI, United States of America; 2 Department of Medicine, University of Michigan, Ann Arbor, MI, United States of America; 3 Henry Ford Malcomb Hospital, Wyndott, MI, United States of America; 4 Beaumont Health System, Royal Oak, MI, United States of America; 5 Spectrum Health System, Grand Rapids, MI, United States of America; 6 McLaren Northern Michigan Health System, Traverse City, MI, United States of America; 7 Detroit Medical Center, Detroit, MI, United States of America; Providence VA Medical Center, UNITED STATES

## Abstract

**Objective:**

To determine the predictors of periprocedural blood transfusion and the association of transfusion on outcomes in high risk patients undergoing endoluminal percutaneous vascular interventions (PVI) for peripheral arterial disease.

**Methods/Results:**

Between 2010–2014 at 47 hospitals participating in a statewide quality registry, 4.2% (n = 985) of 23,273 patients received a periprocedural blood transfusion. Transfusion rates varied from 0 to 15% amongst the hospitals in the registry. Using multiple logistic regression, factors associated with increased transfusion included female gender (OR = 1.9; 95% CI: 1.6–2.1), low creatinine clearance (1.3; 1.1–1.6), pre-procedural anemia (4.7; 3.9–5.7), family history of CAD (1.2; 1.1–1.5), CHF (1.4; 1.2–1.6), COPD (1.2; 1.1–1.4), CVD or TIA (1.2; 1.1–1.4), renal failure CRD (1.5; 1.2–1.9), pre-procedural heparin use (1.8; 1.4–2.3), warfarin use (1.2; 1.0–1.5), critical limb ischemia (1.7; 1.5–2.1), aorta-iliac procedure (1.9; 1.5–2.5), below knee procedure (1.3; 1.1–1.5), urgent procedure (1.7; 1.3–2.2), and emergent procedure (8.3; 5.6–12.4). Using inverse weighted propensity matching to adjust for confounders, transfusion was a significant risk factor for death (15.4; 7.5–31), MI (67; 29–150), TIA/stroke (24; 8–73) and ARF (19; 6.2–57). A focused QI program was associated with a 28% decrease in administration of blood transfusion (p = 0.001) over 4 years.

**Conclusion:**

In a large statewide PVI registry, post procedure transfusion was highly correlated with a specific set of clinical risk factors, and with in-hospital major morbidity and mortality. However, using a focused QI program, a significant reduction in transfusion is possible.

## Introduction

Although not common, blood transfusion may be given to patients undergoing peripheral vascular endoluminal interventions (PVI)[1] for peripheral arterial disease (PAD), particularly if they are anemic at baseline. The same atherosclerotic disease processes that mandate intervention in this population also increase the risk for post-procedure mortality and other adverse cardiac and vascular events. Presumed cardiovascular disease may also drive transfusion decisions.[2] However, the factors predictive of blood transfusion administration are hard to quantify, given variability in patient symptoms and signs, and physician practice preferences and transfusion thresholds. A recent study of percutaneous coronary interventions (PCI) found that patients who were older, female, and those with hypertension, diabetes, and renal insufficiency were more likely to receive blood transfusions.[3]

Blood transfusions are associated with considerable expense[4] and may contribute to major morbidity.[5–7] Some data suggest that patients with coronary artery disease have improved outcomes with restrictive transfusion[8] and that peripheral vascular disease patients may benefit from limiting transfusions.[9] In a large cohort of PCI patients, transfusion was associated with significantly increased risk of MI, stroke, and death.[3] Examination of our own statewide coronary heart interventional data suggested similar conclusions.[10] Other high cardiovascular risk patient populations have experienced a similar reduced, or no worsened, morbidity and mortality with restrictive as compared with liberal transfusion protocols.[8, 11–13]

Conversely, maintaining a physiologic hemoglobin (HgB) may decrease myocardial stress, due to tachycardia required to maintain tissue oxygenation and possibly reduce secondary myocardial injury. In some studies, lower triggers for blood transfusion in “at risk” populations, such as those with preoperative anemia[14] or reduced cardiac reserve and >500-ml of blood loss[15]^-^[16] has been associated with improved outcomes, including reduced mortality. However, these data are from patients undergoing major surgery, and not less invasive endoluminal procedures. These divergent results suggest blood transfusion may be of benefit in certain populations and not others.

This study was undertaken to evaluate among a broad, modern, real world multihospital and multispecialist collaborative patient population undergoing PAD related PVI for 1) pre-procedure predictors of peri-procedural transfusion and 2) the effect of peri-procedural blood transfusion on morbidity and mortality, measured until discharge.

## Methods

### Study Design

Blue Cross/Blue Shield of Michigan Cardiovascular Consortium-Vascular Interventional Collaborative (BMC2 VIC), a large multicenter state-wide quality improvement registry served as the data source for this study. The BMC2 VIC has been determined to have a “non-regulated” status and has been granted an exemption from requirement of patient consent by the University of Michigan Institutional Review Board. All patient records were de-identified prior to analysis.

### Patient population

The BMC2 VIC is a prospective, multicenter observational registry designed to collect information on patients undergoing vascular interventional procedures in an effort to evaluate outcomes and improve quality. This 47 hospital consortium in Michigan collected prospective data on patients undergoing elective PVI procedures with outcomes recorded to discharge. The details of the BMC2 VIC program have been described previously.[17–20]

All patients undergoing elective, urgent, or emergent PVI between January 2010 and December 2014 were included in the analysis. Patients undergoing primary renal, mesenteric, and carotid artery stenting and those undergoing hybrid open surgical–endoluminal therapy were not included. Other exclusion criteria included age < 18 years or critical missing variables such as nadir HgB, medications, or basic demographics. The first hospitalization was studied for patients with multiple subsequent hospital admissions.

A data form was compiled for each patient, including demographic information, past medical and procedural history, standard pre and post procedural blood and chemistry laboratories, procedural indications (claudication and critical limb ischemia [CLI]), procedural urgency, medication types (e.g. statins, antiplatelet therapies, anticoagulants, anti-hypertensives), technical detail of procedures, and associated complications and mortality if they occurred. Urgent procedures were defined as requiring a procedure within 72 hours, while emergent procedure was defined as requiring a procedure within 12 hours. Preprocedural anemia was defined as a HgB < 12 in women and < 13 gm/dL in men. Coronary artery disease (CAD) was defined as a history of myocardial infarction, percutaneous coronary intervention (PCI) or coronary artery bypass graft (CABG). Cerebrovascular disease (CVD) was defined as a history of stroke or transient ischemic attract (TIA).

Peri-procedural blood transfusion was the exposure variable and the primary outcomes were mortality and major morbidity, measured until discharge, with the median of 24 hours (75% IQR: 0–24). Specific morbidities included: myocardial infarction (MI), defined according to the universal definition;[21] transient ischemic attack (TIA) or stroke; and new onset acute renal failure (ARF) requiring hemodialysis (HD). A vascular access complication was defined as one or more of the following: retroperitoneal hematoma, hematoma at access site, pseudoaneurysm, AV fistula, acute thrombosis, or need for open surgical repair.

Data quality and the inclusion of consecutive procedures are ensured by ad hoc queries, random chart review, and a series of diagnostic routines included in the database conducted by the coordinating center. Twice yearly, sites are visited by a nurse monitor from the coordinating center. All cases associated with severe complications and a randomly selected 5% of cases are audited for accuracy.

### Statistical Analysis

Unadjusted comparisons between patients who received peri-procedural transfusion and who did not receive peri-procedural transfusion were performed using the Chi-square or Fisher’s exact test for categorical variables and two-tailed t-test for continuous variables. Adverse outcomes—death, myocardial infarction, TIA or stroke and new onset ARF requiring HD—were compared between no transfusion and transfusion using the Chi-square test. Length of stay was compared using the two-sided t-test with the log transformed values. The equality of transfusion rate in 2010 and 2014 were tested using the Chi-square test. Association between PRBC unit count and each adverse outcome was assessed by the Cochran-Armitage test for trend.

A logistic regression model was developed to assess the relationship between preprocedural variables and peri-procedural transfusion. In this peri-procedural transfusion model, we considered variables such as baseline patient’s characteristics, medicine usage and other clinically relevant variables (S1 Table). The peri-procedure transfusion model included only preprocedural variables to adjust for the case mix effect. We additionally considered several interaction effect due to inconsistent association observed between univariate analysis and multiple logistic regression. Considered two-way interactions were clopidogrel and heparin usage, diabetes and hyperlipidemia, diabetes and pre-anemia, diabetes and procedure status, diabetes and below knee procedure, and diabetes and aorta-iliac procedure and transfusion. A three way interaction of diabetes, aorta-iliac and procedure status is also considered. The stepwise method based on Akaike information criteria (AIC) was applied for variable selection. The Hosmer-Lemeshow p-value and area under a receiver operating characteristic curve (AUC) were reported as model assessment measures for model calibration and discrimination performance, respectively. We also calculated the risk adjusted (or expected) number of peri-procedural transfusions by year and hospital separately. The adjusted numbers were compared with the observed number of peri-procedural transfusions of the corresponding year or hospital. The comparison was expressed in a form of ratio (observed to expected number) and its 95% confidence interval was provided. The prediction ability of the model was validated by 10-fold cross validation method[22] using AUC calculated on the test data (denoted as test AUC).

To assess the relationship between peri-procedural transfusion and other adverse outcomes (death, MI, TIA or stroke, and ARF necessitating HD), we used the inverse probability of treatment weighted (IPTW) method based on propensity score for peri-procedural transfusion. A propensity score model for peri-procedural transfusion was developed using a non-parsimonious model including the preprocedural variables in S1 Table, the year of procedure, and a procedural variable—total IV contrast dose (mL). To rule out confounding due to pre-procedural hemoglobin, we also changed the pre-procedural anemia to the pre-procedural Hemoglobin variable (centered) plus a squared term of this variable in the propensity score model. As total IV contrast dose had additional missing values, the total number of patients included in the IPTW analysis was 22593. The model assessment measures showed robust fitting of the model with Hosmer-Lemeshow p-value = 0.417 and AUC = 0.874. The balance of patient characteristics after adjusted by IPTW were assessed using the Chi-square test and t-test for categorical and continuous variables, respectively. S2 Table shows the adjusted population’s distribution for all the included variables in the propensity score model. All variables showed good balance between transfused and non-transfused cohort except pre-procedural warfarin and heparin use. After applying IPTW using predicted probability from the propensity score model, a logistic regression model was constructed for each adverse outcome to access the association with the peri-procedural transfusion variable. In each adverse outcome model, post-procedural variables such as vascular access complication and heparin use were included. Pre-procedural warfarin and heparin use was also included to adjust for the imbalance observed after applying IPTW. We presented the odds ratio of peri-procedural transfusion variable (exponential of the coefficient and 95% CI) for each adverse outcome.

Nadir hemoglobin has been shown to play an important role in the decision to transfuse and the adverse outcomes due to transfusion in past studies. To control this confounding factor, we conducted a stratification analysis by dividing our data into two groups, nadir hemoglobin < 8 gm/dl and ≥ 8 gm/dl. In each subgroup, the IPTW method was utilized to study the relationship between peri-procedural transfusion and the adverse outcomes adjusting for potential confounders. To control for multiple comparisons across subgroups inflating type I error, the Bonferroni method was employed to calculate appropriate confidence intervals and P-values. Since the nadir hemoglobin introduced more missing values, the total number of patients included in the stratification analysis was reduced to 14,583.

#### Sensitivity Analysis

Unmeasured confounding is an important issue in observational studies since we cannot account for all confounders. To address this issue, we employed a sensitivity analysis to evaluate the robustness of the significant association found between peri-procedural transfusion and the outcomes to a potential unobserved confounder. This method works by specifying the odds ratio of the relationship between unobserved confounder and peri-procedural transfusion and the odds ratio of the relationship between unobserved confounder and outcomes to quantify the strengths of the association between a potential unobserved confounder and peri-procedural transfusion and outcomes. These odds ratios were estimated by the “strongest” odds ratios between the observed confounders and peri-procedural transfusion/ outcomes. Reasonable values of prevalence of the unobserved confounder among no transfusion group were also specified. Bias-adjusted odds ratio (95% CI) of the relationship between peri-procedural transfusion and the outcomes, adjusted for both measured and a potential unmeasured confounder.

All calculations were performed using a statistical software R version 3.0.2.

## Results

### Patient demographics and associations with perioperative transfusion

A total of 39,389 patient visits met the inclusion criteria, and exclusions were due to: multiple hospitalizations (N = 12,741), hybrid procedures (N = 2,245), or missing critical variables (N = 1,108). A total of 23,273 patient procedures were included in the dataset for analysis.

A total of 4.2% (N = 985) patients received a peri-procedural blood transfusion **(Table 1)**, and of these, 93.5% (N = 921) were given post procedurally. Patients who received transfusion had multiple baseline differences as compared to those not receiving a transfusion. Transfused patients were more likely to be older, females, African-Americans, and those with a lower BMI. Of note, non-smokers were more likely to be transfused than current smokers.

**Table 1:** Baseline characteristics of patients not receiving peri-procedural transfusion (No Transfusion) vs. patients who received transfusion (Transfusion).

	No Transfusion (n = 22288)	Transfusion (n = 985)	P-value
Age in years (SD)^†^	68.3 (11.4)	71.5 (12.2)	< .001
Body Mass Index (SD)^†^	28.5 (6.2)	27.4 (6.4)	< .001
Female	9136 (41)	572 (58.1)	< .001
Race			0.027
White	16889 (75.8)	711 (72.2)	
Black	4666 (20.9)	241 (24.5)	
Other	733 (3.3)	33 (3.4)	
Smoking Status			< .001
Never	4153 (18.6)	254 (25.8)	
Former	10084 (45.2)	470 (47.7)	
Current	8051 (36.1)	261 (26.5)	
Pre procedural Anemia^¶^	8851 (39.7)	820 (83.2)	< .001
Family History of Premature CAD	4451 (20)	213 (21.6)	0.219
Hyperlipidemia	19056 (85.5)	775 (78.7)	< .001
Hypertension	20222 (90.7)	920 (93.4)	0.005
Diabetes Mellitus	10739 (48.2)	525 (53.3)	0.002
Prior Congestive Heart Failure (CHF)	4449 (20)	396 (40.2)	< .001
Significant Valve Disease	1419 (6.4)	126 (12.8)	< .001
Chronic Lung Disease (COPD)	6269 (28.1)	361 (36.6)	< .001
CVD or TIA	6178 (27.7)	372 (37.8)	< .001
History of Coronary Artery Disease	12937 (58)	613 (62.2)	0.01
Prior PCI	6786 (30.4)	304 (30.9)	0.809
Previous MI	6189 (27.8)	321 (32.6)	0.001
Previous CABG	5164 (23.2)	230 (23.4)	0.926
Atrial Fibrillation	3007 (13.5)	244 (24.8)	< .001
Other Atherosclerotic Vascular Disease	4459 (20)	212 (21.5)	0.262
Renal Failure CRD	1087 (4.9)	153 (15.5)	< .001
Renal Transplant	215 (1)	14 (1.4)	0.209

Abbreviations: CAD = Coronary Artery Disease, CVD = Cerebrovascular Disease, TIA = Transient Ischemic Attack, PCI = Percutaneous Coronary Intervention, MI = Myocardial Infarction, CABG = Coronary Artery Bypass Graft, AVD = Atherosclerotic Vascular Disease, CRD = Currently Requiring Dialysis. Categorical variables are summarized by No. (%) and p-values are calculated from the Chi-square or Fisher’s exact test.

† (SD) indicate continuous variables with summary measure of mean (standard deviation) and p-value from the student t-test.

¶ Pre Anemia is defined as pre-procedural hemoglobin level < 12 for female and < 13 for male.

Medical conditions in patients that were associated with receiving a blood transfusion included preprocedural anemia, hypertension, diabetes mellitus, congestive heart failure, significant valvular disease, chronic lung disease, coronary artery disease, current GI bleed, atrial fibrillation, history of CVA or TIA, and renal failure requiring hemodialysis. Hyperlipidemia was less common in transfused patients.

Of common medications prescribed to vascular disease patients, statin, warfarin, and preprocedural heparin were more often associated with a peri-procedural blood transfusion, whereas prescription of ASA, beta blockade, and ACEI was less often associated with transfusion **(Table 2).**

**Table 2:** Medicines given pre-procedurally, indication, procedure status and anatomical location for patients who did not received peri-procedural transfusion (No Transfusion) vs. patients who received transfusion (Transfusion).

	No Transfusion (n = 22288)	Transfusion (n = 985)	P-value
*Pre-procedure Medicine*			
Aspirin	18082 (81.1)	750 (76.1)	< .001
Clopidogrel	9822 (44.1)	434 (44.1)	1
Prasugrel	303 (1.4)	7 (0.7)	0.11
Warfarin / Coumadin	2009 (9)	160 (16.2)	< .001
Beta Blockade	10394 (46.6)	385 (39.1)	0.01
Ace Inhibitor	16052 (72)	652 (66.2)	< .001
Statin	2009 (9)	160 (16.2)	< .001
Heparin	1373 (6.2)	256 (26)	< .001
*Indication*			
Claudication	17887 (80.3)	551 (55.9)	< .001
Critical Limb Ischemia†	10149 (45.5)	764 (77.6)	< .001
*Procedure Status*			< .001
Elective	20115 (90.3)	590 (59.9)	
Urgent‡	2003 (9)	308 (31.3)	
Emergent‡	170 (0.8)	87 (8.8)	
*Anatomical Location*			
Aorta—Iliac	7292 (32.7)	314 (31.9)	0.607
Femoral—Popliteal	14334 (64.3)	659 (66.9)	0.103
Below Knee	5468 (24.5)	358 (36.3)	< .001

No. (%) is used as a summary measure. P-values are calculated from the Chi-square test. Contraindicated patients are considered as not taking the medicine.

† Critical Limb Ischemia includes rest pain and Ulcer / Gangrene.

‡ Urgent procedure is defined as a required procedure within 72 hours, but less than 12 hours of symptoms. Emergent procedure is defined as a required procedure within 12 hours of symptoms.

Patients receiving a peri-procedure transfusion were more likely to have CLI and less likely to have claudication as an indication for the PVI, as well as have an more urgent and emergent as compared with elective status. The anatomical location also was associated with transfusion; with below the knee interventions more often associated with transfusion **(Table 2)**.

### Relationship between transfusion and outcomes stratified by preprocedural anemia and nadir hemoglobin <8 and ≥8

Preprocedural anemia is common in elderly patients. Given that this may impact the ordering of a transfusion, we stratified our patient cohort by whether or not the patient was anemic or not. This analysis showed that transfusion was associated with worsened outcomes whether or not they were anemic, including with adjustment by IWPA **(Table 3)**.

**Table 3:** Association between peri-procedural transfusion and adverse outcomes after application of the inverse probability of treatment weights stratified by Pre-procedural Anemia.

*Adverse Outcomes*	*Pre-procedural Anemia*		*No Pre-procedural Anemia*	
	*Odds Ratio (95% CI)*	*P-value*	*Odds Ratio (95% CI)*	*P-value*
Death	6.8 (3.3, 14.2)	<0.001	20.1 (3.2, 125.1)	<0.001
Myocardial Infarction	15 (5.8, 38.7)	<0.001	59.3 (10.1, 350)	<0.001
TIA or Stroke	6.4 (1.2, 34.3)	0.020	7.2 (0.4, 122.2)	0.444
New Requirement for Dialysis	11.6 (2.9, 46.7)	<0.001	12.9 (1.3, 127.3)	0.018

Abbreviations: TIA = Transient Ischemic Attack, CI = Confidence Interval. The odds ratios are estimated from a logistic regression adjusting for post-procedure variables such as vascular access. P-values and CI’s are calculated with a Bonferroni correction to account for multiple comparisons. Pre Anemia is defined as pre-procedural hemoglobin level < 12 for female and < 13 for male.

Nadir hemoglobin, measured in 14,583 patients, was significantly different between transfusion and non-transfusion patients (7.8 ± 1.3 vs. 11.5 ± 1.9 gm/dl, p<0.001). After stratification, a total of 6% (N = 879) patients were in the nadir hemoglobin < 8 gm/dl group, and of these patients, 66% (N = 580) received a peri-procedural blood transfusion. A total of 94% (N = 13,704) patients were in the nadir hemoglobin ≥ 8 gm/dl group, and of these patients, 2.6% (N = 360) received a peri-procedural blood transfusion.

In nadir hemoglobin < 8 gm/dl subgroup, unadjusted rates of death and MI were significantly greater in patients receiving peri-procedural transfusion as compared to those not receiving a transfusion. Unadjusted rates of TIA/stroke and new onset RF with requirement for HD were not significantly different between patients with and without peri-procedural transfusion. In nadir hemoglobin ≥ 8 gm/dl subgroup, unadjusted rates of death, MI, and new onset RF with requirement for HD were significantly greater in patients receiving peri-procedural transfusion as compared to those not receiving a transfusion. The unadjusted rate of TIA/stroke was not significantly different between patients with and without peri-procedural transfusion (S3 Table).

After applying the IPTW method in each subgroup, peri-procedural blood transfusion was independently associated with death and MI in nadir hemoglobin < 8 gm/dl subgroup. In nadir hemoglobin ≥ 8 gm/dl subgroup, peri-procedural blood transfusion was still independently associated with death, MI, and new onset RF with requirement for HD **(Table 4)**.

**Table 4:** Association between peri-procedural transfusion and adverse outcomes after application of the inverse probability of treatment weights stratified by Nadir hemoglobin < 8 (gm/dl) and Nadir hemoglobin ≥ 8 (gm/dl).

*Adverse Outcomes*	*Nadir Hemoglobin < 8 (gm/dl)*		*Nadir Hemoglobin ≥ 8 (gm/dl)*	
	*Odds Ratio (95% CI)*	*P-value*	*Odds Ratio (95% CI)*	*P-value*
Death	1.4 (0.7, 3.0)	1.000	12.5 (3.1, 50.1)	<0.001
Myocardial Infarction	3.4 (1.0, 11.1)	0.036	35 (8.8, 139.5)	<0.001
TIA or Stroke	0.5 (0.1, 2.3)	1.000	4.7 (0.4, 62.2)	0.793
New Requirement for Dialysis	2.9 (0.4, 22.5)	1.000	16.1 (1.5, 170.1)	0.010

Abbreviations: TIA = Transient Ischemic Attack, CI = Confidence Interval. The odds ratios are estimated from a logistic regression adjusting for post-procedure variables such as vascular access. P-values and CI’s are calculated with a Bonferroni correction to account for multiple comparisons.

### Independent predictors of peri-procedural blood transfusion

After the stepwise selection, independent factors that were significantly associated with increased receipt of blood transfusion are female gender, low creatinine clearance (< 60), preprocedural anemia, family history of CAD, CHF, COPD, CVD or TIA, renal failure requiring HD, warfarin, heparin, CLI, aorto-iliac, below knee segment treatments, and an urgent or emergent status **(Table 5)**. Hyperlipidemia and claudication showed significant decreased receipt of blood transfusion. There were three significant interaction effects. First, patients who were prescribed with clopidogrel showed higher increasing trend of receiving transfusion by heparin usage compared to those who were not prescribed to clopidogrel. Similarly, increment of the transfusion receipt from elective to urgent procedure is higher for diabetes patients compared to non-diabetes patients. Lastly, diabetes patients showed less increment of receiving transfusion when the procedure was aorta-iliac segment compared to non-diabetes patients. The model showed good prediction performance with AUC of 0.843, and the 10-fold cross validation showed robust prediction performance on new data sets with average (standard deviation) of test AUC being 0.839 (0.03).

**Table 5:** Selected independent predictors (pre-procedure) of peri-procedural transfusion after applying the AIC stepwise logistic regression.

Predictors	Odds Ratio (95% Confidence Interval)	P-value
Gender (ref. = Male)		
Female	1.9 (1.6, 2.1)	< .001
Age	1 (1, 1)	0.067
Creatinine Clearance (ref. = level > = 60)		
Level < 60	1.3 (1.1, 1.6)	0.002
Pre Anemia	4.8 (4, 5.8)	< .001
Family history of CAD	1.3 (1.1, 1.5)	0.006
Hyperlipidemia	0.8 (0.6, 0.9)	0.003
Diabetes	0.8 (0.7, 1)	0.066
Prior Congestive Heart Failure	1.4 (1.2, 1.6)	< .001
Significant Valve Disease	1.2 (1, 1.5)	0.06
Chronic Lung Disease (COPD)	1.2 (1.1, 1.4)	0.006
CVD or TIA	1.2 (1.1, 1.4)	0.004
Renal Failure Requiring Current Dialysis	1.5 (1.2, 1.9)	0.001
Clopidogrel (pre-procedure)	1 (0.9, 1.2)	0.638
Ace Inhibitor (pre-procedure)	0.9 (0.8, 1)	0.06
Warfarin (pre-procedure)	1.2 (1, 1.5)	0.027
Heparin (pre-procedure)	1.8 (1.4, 2.3)	< .001
Claudication	0.8 (0.7, 0.9)	< .001
Critical Limb Ischemia	1.7 (1.5, 2.1)	< .001
Aorta—Iliac	1.9 (1.5, 2.5)	< .001
Femoral–Popliteal	1.1 (1, 1.4)	0.126
Below Knee	1.3 (1.1, 1.5)	0.008
Procedure Status (ref. = Elective)		
Urgent	1.7 (1.3, 2.2)	< .001
Emergent	8.4 (5.6, 12.4)	< .001
Clopidogrel:Heparin	1.5 (1.1, 2.1)	0.012
Diabetes:Status–urgent	1.4 (1.1, 2)	0.023
Diabetes: Status–emergent	1 (0.6, 2)	0.892
Diabetes:Aorta—Iliac	0.7 (0.5, 1)	0.044

Abbreviations: ref. = reference category, CVD = Cerebrovascular Disease, TIA = Transient Ischemic Attack. All variables are categorical and their reference category is “No” unless the reference category is indicated separately. Hosmer-Lemeshow p-value = 0.636, Area under the ROC curve (AUC) = 0.843

### Hospital transfusion thresholds and related outcomes

**Fig 1:**
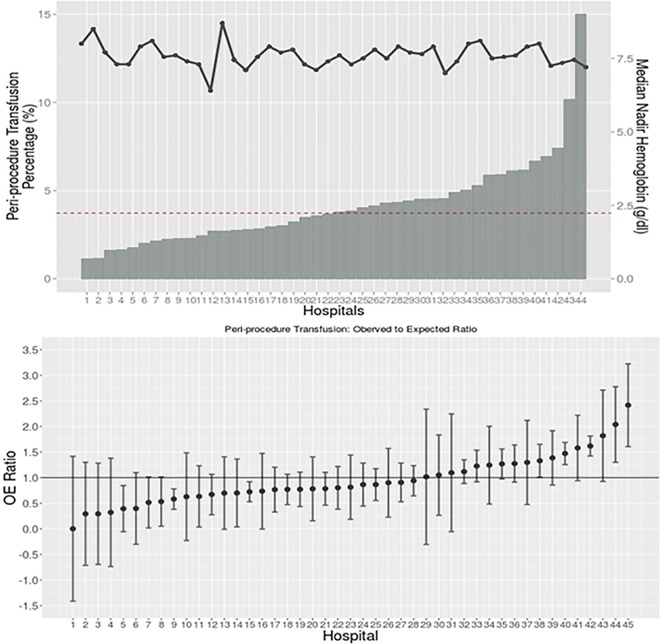
Hospital-wise peri-procedural transfusion distribution. Hospitals are blinded, and both plots may have different order. Two hospitals that had no transfusion were omitted in the both plots. (a) shows unadjusted rate of the transfusion and median nadir hemoglobin level among transfused patients in each BMC2 participating hospitals (blinded). The red dotted horizontal line is showing the median of peri-procedural transfusion rate (3.7%). (b) shows adjusted hospital-wise transfusion distribution using the developed risk model. A horizontal line is at OE ratio = 1.

Unadjusted transfusion rates varied between hospitals, with a 15 fold variation (0% to 15%), and a median transfusion rate of 3.8% (Fig 1). The median nadir HgB among transfused patients in each hospital varied from 6.4 to 8.7 gm/dL, with a median of 7.6 gm/dL. With adjustment, less transfusion rate variation was observed between hospitals as shown with observed to expected (O/E) ratios. Nevertheless, 3 hospitals had transfusion rates significantly less than expected, while 4 hospitals transfused at rates greater than expected.

Among the study population, unadjusted rates of all considered adverse outcomes—death, MI, TIA/stroke, new onset RF with requirement for HD, vascular access complication and length of stay—were significantly greater in patients receiving peri-procedural transfusion as compared to those not receiving a transfusion **(Table 6)**.

**Table 6:** Unadjusted in hospital outcomes comparing patients receiving and not receiving transfusion.

	No Transfusion (n = 22288)	Transfusion (n = 985)	P-value
Death	51 (0.2%)	77 (7.8%)	< .001
Myocardial Infarction	29 (0.1%)	56 (5.7%)	< .001
TIA or Stroke	15 (0.1%)	10 (1%)	< .001
New Requirement for Dialysis	12 (0.1%)	23 (2.3%)	< .001
Length of Stay†	3 (5.1)	12.5 (10)	< .001

Abbreviations: TIA = Transient Ischemic Attack, CI = Confidence Interval. No. (%) is used as a summary measure. P-values are calculated from the Chi-square test.

† Length of Stay is treated as continuous variable, and summarized by mean (standard deviation). P- value is calculated from the t-test on log transformed values.

In the inverse probability of treatment weighted (IPTW) analysis, peri-procedural blood transfusion was independently associated with death, MI, TIA/stroke, and New Requirement for Dialysis after adjusting for pre-procedural warfarin and heparin use, post-procedural vascular access complication, and post-procedural heparin use **(Table 7).** We also found an association with the number of PRBC units transfused and the outcomes of death, MI, and vascular access complication **(Table 8).**

**Table 7:** Association between peri-procedural transfusion and adverse outcomes after application of the inverse probability of treatment weights.

*Adverse Outcomes*	*Odds Ratio (95% CI)*	*P-value*
Death	15.4 (7.5, 31.4)	<0.001
Myocardial Infarction	66.8 (29.6, 150.6)	<0.001
TIA or Stroke	24 (7.9, 73.4)	<0.001
New Requirement for Dialysis	18.9 (6.2, 57.1)	<0.001

Abbreviations: TIA = Transient Ischemic Attack, CI = Confidence Interval. The odds ratios are estimated from a logistic regression adjusting for post-procedure variables such as vascular access complication, post-procedure Heparin use and age.

**Table 8:** Adverse outcome frequency and percentage by PRBC count.

*PRBC*	*Total*	*Death*	*MI*
1	145	6 (4.1%)	4 (2.8%)
2	252	15 (6.0%)	9 (3.6%)
3	56	2 (3.6%)	5 (8.9%)
4	59	7 (11.9%)	5 (8.5%)
5	25	4 (16%)	2 (8%)
≥ 6	38	8 (21.1%)	7 (18.4%)
P-value		< .001	< .001

PRBC count records have been collected since 2012. For patients who had both in-lab and post procedure transfusion, sum of PRBC is used. Outcomes are at discharge only. P-values are calculated using Cochran-Armitage trend test.

### Relationship between transfusion and outcomes adjusted for both measured and potential unmeasured confounders

Results from sensitivity analysis showed a robust association between peri-procedural blood transfusion and death, MI, TIA/stoke, and New Requirement for Dialysis, after adjusting for both measured and a potential unmeasured confounder (S4 Table). We specified three reasonable values of prevalence of the unobserved confounder among no transfusion group (10%, 20%, and 30%). Within the range of the prevalence of unobserved confounder among no transfusion group we specified, if the prevalence of unobserved confounder went higher, the effect of the potential unobserved binary confounder on the relationship between peri-procedural transfusion and adverse outcomes went lower, so the odds ratio of the relationship between peri-procedural transfusion and adverse outcomes went higher, and closer to the odds ratio without adjustment of unobserved confounder.

### Focused QI Efforts to Reduce Peri-Procedural Transfusion

**Fig 2:**
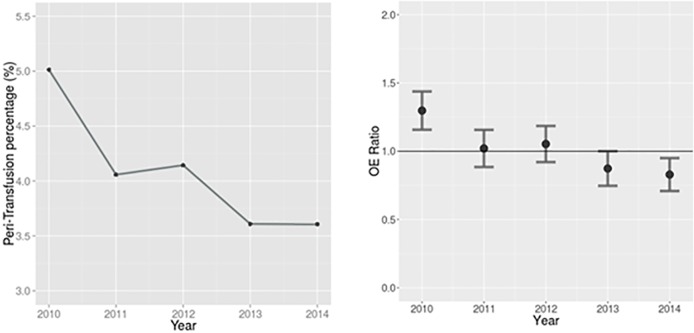
Annual trend of peri-procedural transfusion. (a) shows unadjusted percentage of the transfusion, and there was 28% decrease in the transfusion rate from 2010 to 2014 (5% to 3.6%, p = 0.001). (b) shows adjusted transfusion trend using the developed risk model. A horizontal line is at OE ratio = 1. OE ratio in 2010 and 2014 were 1.3 (95% CI: 1.2–1.4) and 0.83 (0.71–0.95), respectively.

The 2008–2010 transfusion rates were in the 6–7% range across the collaborative. During this period, the BMC2 VIC quality initiative focused on decreasing peri-procedural blood transfusion. To reduce bleeding potential, examples included a weight-based heparin anticoagulation dosing, with an initial heparin dose at < = 60 U/kg, and level of anticoagulation to achieve an ACT of 200 to 250 seconds.[23] The collaborative also issued transfusion guidelines to all participating hospitals and physician leaders with a recommendation not to transfuse patients with HgB ≥ 8 if not symptomatic. Physicians and hospitals were issued reports that detailed conformity with these recommendations and bench marks across the collaborative. By 2014, BMC2 VIC had achieved a 28% decrease in hospital level blood transfusions (Fig 2; p = 0.001 for trend). This trend was also maintained with risk adjustment as shown in the observed to expected ratios per year. The ratio was 1.3 (95% CI: 1.2–1.4) in 2010 and it decreased to 0.83 (0.71–0.95) in 2014. Both of the ratios were significantly different from 1.

## Discussion

Peripheral vascular interventions by their very nature are associated with potential blood loss. In most elective PVI procedures, blood loss is low and transfusions are unnecessary. However, vascular disease is a systemic process and vascular disease patients characteristically have multiple comorbidities, particularly cardiovascular disease, and maybe anemic pre-procedurally.[24, 25] These relevant issues underscore the major findings of this study: 1) blood transfusion is more likely in older, sicker patients; 2) specific factors predict the use of blood transfusion; 3) blood transfusions are associated with increased post-procedural morbidity and mortality; 4) blood transfusion thresholds vary greatly amongst hospitals performing similar procedures; and 5) a focused QI program was associated with significantly decreased receipt of blood transfusion.

While several of the associated factors with transfusion (i.e. preprocedural anemia, GI bleed, older age and multiple comorbidities) were not surprising, several bear mention. First, women were more likely to receive a transfusion. A very similar finding in a large observational study of CABG patients showed females were ~3 fold more likely to be anemic.[25] Similarly, in a large PCI registry, women were also more likely to be anemic[26] and more likely to be transfused.[3] This was also observed in our statewide PCI registry.[10] The physiological reasons for this are not immediately apparent. Secondly, preprocedural heparin and warfarin were independently associated with receipt of blood transfusion. We did not collect preprocedural INR or aPTT, and it is possible patients’ heparin or warfarin associated coagulopathy was not fully reversed, and accounted for greater bleeding.

When controlling for confounders, including stratification by preprocedure anemia and post procedure nadir HgB, we found that receipt of blood transfusion in PVI patients was highly associated with major morbidity and mortality. Indeed, this was proportionate to the number of units received. Blood transfusion can directly suppress immune function, and is potentially thrombogenic.[27] The immunosuppressive effects of PRBC transfusion have been attributed to both a direct immunomodulatory effect of the transfused donor leukocytes as well as induced alterations in the recipients’ circulating leukocytes.[28] Others have shown that complications increase in a dose-dependent fashion with PRBC transfusions.[29–31] The mechanism of how transfusion might increase morbidity and mortality is likely multifactorial and may be related to the number of units transfused–for example, limiting the absolute number of red blood cells transfused (<4 in the cardiac surgery population) may decrease circulating pro-inflammatory cytokines and decrease the risk of infection.[29]

We were surprised to find that receipt of blood transfusion was associated with increased, rather than decreased MI. This association was even observed in those with a nadir HgB < 8 gm/dL. An accepted paradigm is that anemia may directly cause myocardial ischemic damage due to increased myocardial demand secondary to compensatory tachycardia. A recent retrospective study showed greater mortality in a defined restrictive transfusion protocol as compared with liberal transfusion practice in acute coronary syndrome patients.[32] However, not all studies have found this association,[33] and it is likely the acute myocardial ischemia physiology is different than chronic CAD. Supporting this contention are two large RCT studies that suggest at risk patients with cardiovascular disease have no increased incidence of cardiovascular morbidity or mortality with restrictive transfusion.[8, 34] Lastly, our data suggest that a transfusion for a nadir HgB < 8 gm/dL is not associated with death, TIA or stroke, or new need for HD, while those receiving a transfusion with a HgB > 8 was associated with death and major morbidity.

Whether correction of preprocedural anemia without transfusion could improve outcomes is not clear. The American College of Physicians recommends against erythropoiesis-stimulation agents, but intravenous iron may be efficacious.[35] Probably more important is determining the etiology of anemia in these patients, such as malignancy or chronic infection, which may be treatable.

As other studies[8, 34, 36] suggest no patient harm with a restrictive transfusion policy, the wide ranging transfusion thresholds amongst the 44 hospitals suggests markedly different practices. Indeed, these variations are often at the practitioner level.[37] We are limited in knowing the specific indications for transfusion at an individual center, and it may be that certain hospitals treat more urgent cases or procedures that are likely to be associated with transfusion. With risk adjustment, most hospitals were within the expected number of transfusions–yet several were significantly higher than expected suggesting that factors beyond patient mix explain this variation.

In 2009, the BMC2 Physician Advisory Committee approved the initiation of a QI effort focused on reducing transfusion across sites to < 7%, with a transfusion threshold of 8 g/dL. The QI team found most hospitals had no transfusion protocols and significant site variation. Using an interventional education approach, we disseminated best practices, including recommendations about the use of peri-procedural heparin, groin edicate, and judicious use of blood. A Quality Improvement Team was created comprised of BMC2 leadership and a plan was implemented to visit hospitals where transfusion rates were low in 2010. A detailed questionnaire was developed about site processes for PVI and transfusion. The quality improvement worked together to merge the best protocols and order sets into a comprehensive set of “best practices” for the collaborative. Although we cannot say this intervention was the sole cause for the decreased transfusion rate over time, the dramatic decline in transfusion over the study period is noteworthy; from ~ 7% in 2009 to 3.6% in 2014. Others have documented a similar benefit with a focused statewide collaborative initiative.[13]

Limitations of this study include the retrospective nature of the analysis. We cannot account for all cofounders (such as intra- or post-procedural fluid management, interventionalists’ technical skill, bone marrow failure, or procedural blood loss quantification; all of which were outside the registry data), despite rigorous standardized prospective collection and assessment of data and statistical adjustments. However, we have captured many of the variables in prospective studies[8, 34] and have the advantage of peri-procedural medication documentation, and rigorous consistent definitions amongst hospitals in the consortium. We also note the transfusion related outcomes are associations, and do not prove causation. We acknowledge that although the patient population is large, the event rates were relatively low, and the confidence intervals around the OR large. However, comparison of alternative variables and models of propensity matching yielded similar estimates with reasonable standard errors (data not shown). We only had limited data regarding the timing of the complication in relation to the timing of blood transfusion, and anticoagulation intensity (via ACT) was available in only about 50% of the population. However, the correlations are quite strong statistically after applying the inverse probability treatment weight (IPTW), and consistent with other studies in the literature.[3] Regarding the performance of IPTW, a study showed when compared to the matching method, the IPTW approach features larger variance, but smaller bias for estimated absolute risk reduction^39^. We chose the IPTW method, since bias reduction was more important in our study. Furthermore, within our database, we don’t have a variable that affects the outcomes only through transfusion, so we were unable to use an instrumental variable analysis technique. A falsification endpoint is an outcome that is influenced by unobserved confounder of the causal effect study, but not directly influenced by the treatment/exposure.[38] We did not believe that we had a falsification endpoint that was affected by unmeasured confounders but not affected by transfusion in this dataset, so we were not able to perform a falsification endpoint analysis as other studies have used.

Although our data do not allow us to comment on transfusion thresholds, consensus guideline recommendations,[32] and a recent large transfusion threshold trial in septic patients,[36] suggest that restrictive transfusion practice in (HgB 7–8 gm/dL) is a reasonable strategy unless the patient is symptomatic or has active hemorrhage. More importantly, these findings strongly suggest the need for a prospective RCT comparing a restrictive transfusion practice (perhaps HgB ~7.0) vs. a liberal transfusion practice (HgB~ 9) in vascular procedural patients, who often have very significant atherosclerotic comorbidities.

## Supporting Information

S1 Table Considered predictors in the logistic regression model for peri-operative transfusion and propensity score model. (DOC)

S2 Table Baseline characteristics of patients who did not receive a peri-procedural transfusion (No Transfusion) vs. patients who received transfusion (Transfusion) after application of inverse probability weights. (DOCX)

S3 Table Unadjusted in hospital outcomes comparing patients receiving and not receiving transfusion stratified by nadir hemoglobin < 8 (gm/dl) and nadir hemoglobin ≥ 8 (gm/dl). (DOCX)

S4 Table Association between peri-procedural transfusion and adverse outcomes (Odds Ratio with 95% CI), adjusted for both observed confounders and potential unobserved binary confounder using sensitivity analysis method. (DOCX)
