# Supplementary material for: The Association of Peri-Procedural Blood Transfusion with Morbidity and Mortality in Patients Undergoing Percutaneous Lower Extremity Vascular Interventions: Insights from BMC2 VIC
Source: PLoS One. 2016 Nov 11;11(11):e0165796. doi: 10.1371/journal.pone.0165796 (PMC5106007; doi:10.1371/journal.pone.0165796)
Supplement: S1 Table — (DOC) [file pone.0165796.s001.doc]

**Table S1.**

| ***Patient Information*** | | | | | | | | |
| --- | --- | --- | --- | --- | --- | --- | --- | --- |
| Age (continuous) | Gender (Female/Male*) | | | | Race (White*/Black/Other) | | | |
| Body Mass Index (High>30/Normal*/Low<18), continuous in propensity score model | | | | | | | | |
| Smoking Status (Never*/Former/Current) | | | | |  | | | |
| ***Patient History (Yes/No*)*** | | | | | | | | |
| Pre-operative Anemia (Centered Pre-operative Hemoglobin (g/dl) and Centered Pre-operative Hemoglobin^2^ in propensity score model) | | | | | | | | |
| Diabetes Mellitus | | | | | Hyperlipidemia | | | |
| Hypertension | | | | | Prior Congestive Heart Failure | | | |
| Significant Valve Disease | | | | | History Of Coronary Artery Disease | | | |
| Prior Percutaneous Coronary Intervention | | | | | Previous Myocardial Infarction | | | |
| Prior Coronary Artery Bypass Graft | | | | | Other Atherosclerotic Vascular Disease | | | |
| Renal Failure requiring Current Dialysis | | | | | Current GI Bleed | | | |
| Atrial Fibrillation | Family History of Premature Coronary Artery Disease | | | | | | | |
| Chronic Lung Disease | Cerebrovascular Disease Or Transient Ischemic Attack | | | | | | | |
| ***Pre-operative Medicine (Yes/No*)*** | | | | | | | | |
| Aspirin | Clopidogrel | | | | Beta Blocker | | | Ace Inhibitor |
| Statin | Warfarin | | | | Heparin | | |  |
| ***Others*** | | | | | | | | |
| Pre-procedure Creatinine Clearance (High≥60*/Low<60) | | | | | | | | |
| Anatomical Locations: | | Aorta-Iliac | | Femoral - Popliteal | | | Below Knee | |
| Procedure Status (Elective*/Urgent/Emergent) | | | | | | | | |
| ***Two-way interaction terms included in the final model*** | | | | | | | | |
| Clopidogrel:Heparin | | | Diabetes:Procedure Status | | | Diabetes:Aorta-Iliac procedure | | |
| ***Only included in the propensity score model*** | | | | | | | | |
| Procedure Year | | | | Total IV contrast dose (mL) | | | | |

Parenthesis indicate used category for each variable, and * indicate reference category. Continuous variable is indicated as continuous. Patient history and pre-operative medicine have the same category.
