# Supplementary material for: The Association of Peri-Procedural Blood Transfusion with Morbidity and Mortality in Patients Undergoing Percutaneous Lower Extremity Vascular Interventions: Insights from BMC2 VIC
Source: PLoS One. 2016 Nov 11;11(11):e0165796. doi: 10.1371/journal.pone.0165796 (PMC5106007; doi:10.1371/journal.pone.0165796)
Supplement: S2 Table — (DOCX) [file pone.0165796.s002.docx]

**Table S2.**

|  | No Transfusion  (n= 21643) | Transfusion  (n= 950) | P-value |
| --- | --- | --- | --- |
| Age in years (SD)^†^ | 68 (11.5) | 69 (11.6) | 0.508 |
| Body Mass Index (SD)^†^ | 28 (6.2) | 28 (6.1) | 0.679 |
| Female | 9070 (41.9) | 416 (43.8) | 0.580 |
| Race |  |  | 0.455 |
| White | 16330 (75.5) | 746 (78.6) |  |
| Black | 4579 (21.2) | 172 (18.1) |  |
| Other | 734 (3.4) | 32 (3.4) |  |
| Smoking Status |  |  | 0.860 |
| Never | 4128 (19.1) | 168 (17.6) |  |
| Former | 9808 (45.3) | 439 (46.2) |  |
| Current | 7707 (35.6) | 343 (36.1) |  |
| Centered Pre Hemoglobin (SD)^†^ | -0.02 (2) | 0.09 (2.2) | 0.509 |
| Centered Pre Hemoglobin^2^ (SD)^†^ | 4 (5.5) | 4.7 (5.5) | 0.080 |
| Procedure Year |  |  | 0.459 |
| 2010 | 3932 (18.2) | 148 (15.6) |  |
| 2011 | 4091 (18.9) | 228 (24) |  |
| 2012 | 4393 (20.3) | 189 (19.9) |  |
| 2013 | 4518 (20.9) | 185 (19.5) |  |
| 2014 | 4709 (21.8) | 200 (21) |  |
| Family History of Premature CAD | 4378 (20.2) | 558 (17.5) | 0.254 |
| Hyperlipidemia | 18454 (85.3) | 816 (85.9) | 0.759 |
| Hypertension | 19694 (91) | 843 (88.7) | 0.422 |
| Diabetes Mellitus | 10490 (48.5) | 473 (49.8) | 0.701 |
| Prior Congestive Heart Failure (CHF) | 4539 (21) | 202 (21.3) | 0.888 |
| Significant Valve Disease | 1447 (6.7) | 68 (7.1) | 0.772 |
| Chronic Lung Disease (COPD) | 6182 (28.6) | 290 (30.6) | 0.504 |
| CVD or TIA | 6122 (28.3) | 299 (31.5) | 0.302 |
| History of Coronary Artery Disease | 12609 (58.3) | 558 (58.8) | 0.887 |
| Prior PCI | 6598 (30.5) | 274 (28.8) | 0.591 |
| Previous MI | 6050 (28) | 262 (27.6) | 0.914 |
| Previous CABG | 5031 (23.2) | 215 (22.6) | 0.830 |
| Current GI Bleed | 370 (1.7) | 16 (1.6) | 0.885 |
| Atrial Fibrillation | 3030 (14) | 147 (15.5) | 0.405 |
| Other Atherosclerotic Vascular Disease | 4308 (19.9) | 209 (22) | 0.444 |
| Renal Failure CRD | 1166 (5.4) | 57 (6) | 0.474 |
| Procedure Status |  |  | 0.058 |
| Elective | 19242 (88.9) | 820 (86.3) |  |
| Urgent‡ | 2124 (9.8) | 117 (12.3) |  |
| Emergent‡ | 277 (1.3) | 13 (1.4) |  |
| Low Creatinine Clearance (< 60) | 8031 (37.1) | 407 (42.9) | 0.078 |
| Total IV contrast dose (SD) ^†^ | 153 (86.4) | 157 (93.8) | 0.517 |
| *Pre-procedure Medicine* |  |  |  |
| Aspirin | 17567 (81.2) | 769 (80.9) | 0.921 |
| Clopidogrel | 9589 (44.3) | 403 (42.4) | 0.598 |
| Warfarin / Coumadin | 2033 (9.4) | 138 (14.5) | 0.013 |
| Beta Blockade | 13294 (61.4) | 574 (60.4) | 0.779 |
| Ace Inhibitor | 10060 (46.5) | 426 (44.9) | 0.652 |
| Statin | 15561 (71.9) | 691 (72.7) | 0.779 |
| Heparin | 1536 (7.1) | 104 (10.9) | 0.001 |
| *Anatomical Location* |  |  |  |
| Aorta - Iliac | 7052 (32.6) | 321 (33.8) | 0.717 |
| Femoral - Popliteal | 13956 (64.5) | 628 (66.1) | 0.636 |
| Below Knee | 5477 (25.3) | 217 (22.8) | 0.313 |
| *Indication* |  |  |  |
| Claudication | 17193 (79.4) | 731 (76.9) | 0.256 |
| Critical Limb Ischemia | 10180 (47) | 477 (50.2) | 0.376 |

Abbreviations: CAD = Coronary Artery Disease, CVD = Cerebrovascular Disease, TIA = Transient Ischemic Attack, PCI = Percutaneous Coronary Intervention, MI = Myocardial Infarction, CABG = Coronary Artery Bypass Graft, AVD = Atherosclerotic Vascular Disease, CRD = Currently Requiring Dialysis

Categorical variables are summarized by No. (%) and p-values are calculated from the Chi-square test.

Model assessment measures: Hosmer-Lemeshow p-value = 0.417, AUC = 0.874

† (SD) indicate continuous variables with summary measure of mean (standard deviation) and p-value from the student t-test.
