# Supplementary material for: The Association of Peri-Procedural Blood Transfusion with Morbidity and Mortality in Patients Undergoing Percutaneous Lower Extremity Vascular Interventions: Insights from BMC2 VIC
Source: PLoS One. 2016 Nov 11;11(11):e0165796. doi: 10.1371/journal.pone.0165796 (PMC5106007; doi:10.1371/journal.pone.0165796)
Supplement: S3 Table — (DOCX) [file pone.0165796.s003.docx]

**Table S3.**

|  | *Nadir Hemoglobin < 8 (gm/dl)* | | | | *Nadir Hemoglobin ≥ 8 (gm/dl)* | | | |
| --- | --- | --- | --- | --- | --- | --- | --- | --- |
|  | *No Transfusion*  *(n = 299)* | *Transfusion*  *(n = 580)* | *Odds Ratio*  *(95% CI)* | *P-value* | *No Transfusion*  *(n = 13344)* | *Transfusion*  *(n = 360)* | *Odds Ratio*  *(95% CI)* | *P-value* |
| Death | 12 (4%) | 54 (9.3%) | 2.5 (1.3, 4.7) | 0.005 | 34 (0.3%) | 20 (5.6%) | 23 (13.1, 40.4) | <0.001 |
| Myocardial Infarction | 5 (1.6%) | 39 (6.7%) | 4.2 (1.7, 10.9) | 0.001 | 24 (0.2%) | 16 (4.4%) | 25.8 (13.6, 49) | <0.001 |
| TIA or Stroke | 3 (1%) | 8 (1.4%) | 1.4 (0.4, 5.2) | 0.758 | 12 (0.1%) | 2 (0.6%) | 6.2 (1.4, 27.8) | 0.051 |
| New Requirement for Dialysis | 2 (0.7%) | 14 (2.4%) | 3.7 (0.8, 16.3) | 0.106 | 8 (0.1%) | 7 (1.9%) | 33.1 (11.9, 91.7) | <0.001 |

Abbreviations: TIA = Transient Ischemic Attack , CI = Confidence Interval

No. (%) is used as a summary measure. P-values are calculated from the Chi-square test or Fisher’s exact test
