# Supplementary material for: The Association of Peri-Procedural Blood Transfusion with Morbidity and Mortality in Patients Undergoing Percutaneous Lower Extremity Vascular Interventions: Insights from BMC2 VIC
Source: PLoS One. 2016 Nov 11;11(11):e0165796. doi: 10.1371/journal.pone.0165796 (PMC5106007; doi:10.1371/journal.pone.0165796)
Supplement: S4 Table — (DOCX) [file pone.0165796.s004.docx]

**Table S4.**

| *Adverse Outcomes* | *Prevalence of U =10%* | *Prevalence of U =20%* | *Prevalence of U =30%* |
| --- | --- | --- | --- |
| Death | 5.1 (2.5, 10.5) | 5.8 (2.9, 11.9) | 6.9 (3.4, 14.1) |
| Myocardial Infarction | 20.8 (9.2, 46.9) | 24.2 (10.7, 54.6) | 29 (12.9, 65.4) |
| TIA or Stroke | 5.6 (1.8, 17) | 7.4 (2.4, 22.5) | 9.4 (3.1, 28.7) |
| New Requirement for Dialysis | 3.6 (1.2, 11) | 5.3 (1.7, 16) | 7 (2.3, 21.1) |

Abbreviations: TIA = Transient Ischemic Attack, CI = Confidence Interval

Prevalence of U is the prevalence of the unobserved confounder among no transfusion group
